# Apparent Opportunities and Hidden Pitfalls: The Conflicting Results of Restoring NRF2-Regulated Redox Metabolism in Friedreich’s Ataxia Pre-Clinical Models and Clinical Trials

**DOI:** 10.3390/biomedicines11051293

**Published:** 2023-04-27

**Authors:** Jessica Tiberi, Marco Segatto, Maria Teresa Fiorenza, Piergiorgio La Rosa

**Affiliations:** 1 Division of Neuroscience, Department of Psychology, Sapienza University of Rome, Via dei Marsi 78, 00185 Rome, Italy; 2 PhD Program in Behavioral Neuroscience, Sapienza University of Rome, Via dei Marsi 78, 00185 Rome, Italy; 3 Department of Bioscience and Territory, University of Molise, Contrada Fonte Lappone, 86090 Pesche, Italy; 4 European Center for Brain Research, IRCCS Fondazione Santa Lucia, Via del Fosso di Fiorano 64, 00179 Rome, Italy

**Keywords:** Friedreich’s ataxia, FRDA, NRF2, ROS, oxidative stress, antioxidants

## Abstract

Friedreich’s ataxia (FRDA) is an autosomal, recessive, inherited neurodegenerative disease caused by the loss of activity of the mitochondrial protein frataxin (FXN), which primarily affects dorsal root ganglia, cerebellum, and spinal cord neurons. The genetic defect consists of the trinucleotide GAA expansion in the first intron of *FXN* gene, which impedes its transcription. The resulting FXN deficiency perturbs iron homeostasis and metabolism, determining mitochondrial dysfunctions and leading to reduced ATP production, increased reactive oxygen species (ROS) formation, and lipid peroxidation. These alterations are exacerbated by the defective functionality of the nuclear factor erythroid 2-related factor 2 (NRF2), a transcription factor acting as a key mediator of the cellular redox signalling and antioxidant response. Because oxidative stress represents a major pathophysiological contributor to FRDA onset and progression, a great effort has been dedicated to the attempt to restore the NRF2 signalling axis. Despite this, the beneficial effects of antioxidant therapies in clinical trials only partly reflect the promising results obtained in preclinical studies conducted in cell cultures and animal models. For these reasons, in this critical review, we overview the outcomes obtained with the administration of various antioxidant compounds and critically analyse the aspects that may have contributed to the conflicting results of preclinical and clinical studies.

## 1. Introduction

Friedreich’s ataxia (FRDA; also known as FA; OMIM, #229300) is an inherited, autosomal, recessive neurodegenerative disorder belonging to the autosomal recessive cerebellar ataxias (ARCAs) group. Neurological manifestations of the disease are mainly caused by the degeneration of dorsal root ganglia (DRG) and cerebellum [1,2]. FRDA is the most common form of inherited ataxia [3,4], affecting approximately 1:20,000–1:50,000 people of the Caucasian population [5,6], while it is very rare in Southeast Asians, in Sub-Saharan Africans, and among Native Americans [7]. The typical onset of the pathology is between 10–15 years of life [6], although 15% of cases, i.e., those arising after the second or the fourth decade of life, are classified as late-onset (LOFA) or very-late-onset (vLOFA) FRDA forms, respectively [8,9,10].

**Figure 1:**
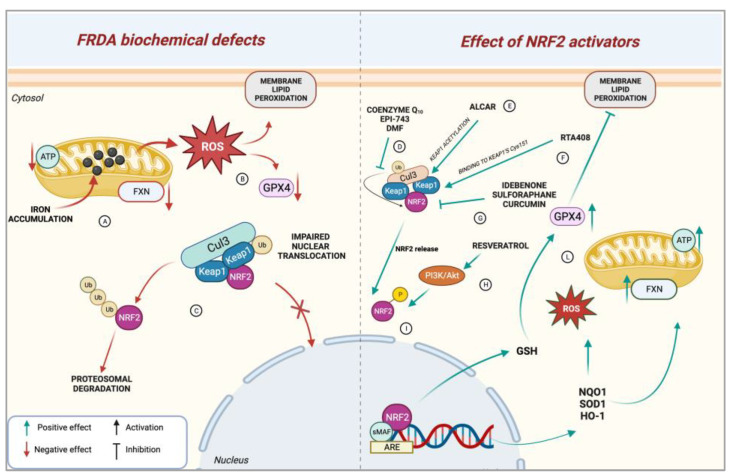
A scheme of biochemical defects of FRDA and how the activation of NRF2-signaling axis can rescue these defects. (**A**) FXN deficiency determines mitochondrial impairments, lower ATP synthesis, iron accumulation, and ROS production. (**B**) High ROS levels lead to lipid peroxidation and lower GPX4 expression. (**C**) The cellular antioxidant defence is impaired due to reduced NRF2 nuclear translocation. (**D**) Coenzyme Q10, EPI-743, and DMF administration prevent NRF2 ubiquitination, inhibiting its degradation. (**E**) ALCAR induces KEAP1 acetylation, destabilizing its interaction with NRF2. (**F**) RTA408 directly binds to KEAP1’s Cys 151, destabilizing its interaction with NRF2. (**G**) Idebenone, sulforaphane, and curcumin interact with KEAP1, weakening its interaction with NRF2. (**H**) Resveratrol induces NRF2 phosphorylation and nuclear translocation through a PI3K/Akt-dependent mechanism. (**I**) In the nucleus, the NRF2–sMAF complex stimulates the transcription of NRF2-target genes, while NRF2-mediated GSH synthesis upregulation promotes GPX4 function. (**L**) This determines the reduction of ROS level, iron accumulation, rescue of FXN expression, and mitochondrial ATP production. Created with @BioRender.

The etiologic determinant of FRDA is the lack of frataxin (FXN), the product of *FXN* gene [11], a 14 kDa mitochondrial protein involved, among additional functions still to be clarified, in iron–sulphur (Fe-S) cluster biosynthesis [12], iron metabolism and transport [13], and antioxidant defence [14]. FXN deficiency determines defective mitochondrial function and Fe-S cluster enzyme activity, reduced adenosine triphosphate (ATP) production, and an increase of reactive oxygen species (ROS) responsible for lipid peroxidation [13,14,15,16,17,18]. The increased production of ROS and oxidative stress [19] caused by FXN deficiency cannot be counterbalanced by the canonical antioxidant response due to the derangement of the nuclear factor erythroid 2-related factor 2 (NRF2) activity [20,21,22,23,24,25], the master regulator of antioxidant defence and redox metabolism. Indeed, decreased FXN levels in the DRG and cerebellum isolated from an FRDA mouse model strictly correlated with a decrease of *Nrf2* transcripts [21], corroborating evidence that weakened antioxidant defence contributes to the higher sensitivity to oxidative insults in FRDA cells. Accordingly, oxidative stress represents one of the main pathophysiological components of FRDA onset and progression [26,27,28,29,30]. This evidence has prompted much of the research in FRDA to focus on this specific component of the disease, conceiving the induction of NRF2 activity as a means to counteract oxidative stress. However, in spite of promising results of preclinical studies showing that defects of FRDA animal models and cells isolated from patients’ biopsies are partially rescued by promoting NRF2 stability and by activating the NRF2 signalling pathway (Figure 1), in most cases, clinical data have yielded conflicting results. Within this framework, in this review, we summarise the current knowledge on FRDA impairment of the NRF2 signalling axis and redox metabolism. Then, we provide a comprehensive overview of the efficacy of past and ongoing antioxidant therapies aimed at inducing NRF2 activation and restoring FXN function both in in vitro and in vivo models and in clinical trials. Finally, we attempt to identify the reasons that underlie the partial improvements in FRDA patients subjected to antioxidants therapies, with the aim of contributing to a better understanding of the potential benefits and limitations of this approach when facing FRDA disease.

## 2. FRDA: Clinical and Molecular Features

Clinically, FRDA manifests as a multisystemic disease mainly characterised by peripheral and central nervous system (CNS) manifestations such as progressive gait and limb ataxia, caused by spinocerebellar degeneration and loss of proprioception, and often associated with impairment of fine motor skills such as handwriting and swallowing [31]. Dysarthria and muscular weakness also manifest as a result of progressive degeneration of DRG, corticospinal tracts, dorsal spinocerebellar tracts of the spinal cord, and cerebellum [32]. Additionally, motor and mental reaction times can be significantly slowed down [33,34], and motor planning is markedly impaired [35]. These neurologic manifestations are accompanied by non-neurological outcomes, including hypertrophic cardiomyopathy [36], which usually represents the primary cause of death [37,38]; scoliosis [39]; diabetes mellitus [40]; optic nerve atrophy; and hearing impairment [41]. Progression of the pathology is primarily assessed through rating scales aimed at evaluating the trend of neurological impairment. To date, three different scales have been developed and validated to evaluate symptoms and disease progression in ataxic disorders in general and/or in FRDA: The International Cooperative Ataxia Rating Scale (ICARS), the Scale for the Assessment and Rating of Ataxia (SARA), and FRDA Rating Scale (FARS) [42,43]. In detail, the ICARS score was primarily developed to assess cerebellar ataxia as a whole [44], but a great deal of evidence supports its use as a powerful tool also in FRDA due to its high inter-rater reliability [31,45,46]. ICARS consists of 19 items grouped in following four subclasses: (i) posture and gait disturbance, (ii) limb ataxia, (iii) dysarthria, and (iv) oculomotor disorders [44]. SARA was first designed to provide a reliable and valuable clinical scale in autosomal dominant ataxias [47]. SARA can be performed rapidly because it is limited to eight items: (i) gait, (ii) stance, (iii) sitting, (iv) speech disturbances, (v) finger chase, (vi) nose-finger test, (vii) fast alternating hand movements, and (viii) heel-shin slide. Similar to ICARS, SARA inter-rater reliability has been demonstrated to be an acceptable means of assessing disease progression in FRDA [43]. The FARS score was specifically developed to address the phenotypic characteristics of FRDA (e.g., prominent sensory dysfunction because of primary degeneration of DRG). The total score is determined by the following assessments: (i) neurologic examination of bulbar, upper-limb, lower-limb, peripheral nerve, and upright stability/gait functions; (ii) functional staging to evaluate overall mobility, speech, and test hand coordination; and (iii) activities linked to daily living [48]. Of note, only FARS includes features not directly related to the physical examination, such as activities of daily living.

In 1996, Campuzano and her team discovered that in 96% of FRDA patients, the genetic cause of the pathology is ascribable to a homozygous guanine-adenine-adenine (GAA) trinucleotide repeat expansion within the first intron of the *FXN* gene, which is located on chromosome 9q21.11 [11]. The remaining 4% of probands harbour one GAA expansion in one allele and a different pathogenic mutation in the other, with 44 point mutations identified to date [49]. The effect of these alterations can be an impairment of the initiation of *FXN* transcription or the occurrence of frameshift and/or missense mutations, which lead to a strong reduction of FXN expression and function [50,51,52]. Different models have attempted to elucidate this phenomenon on a molecular basis. For example, a study demonstrated the presence of a heterochromatic core in the proximity of the GAA triplet repeats and at the *FXN* promoter, which in turn determined a faulty transcription initiation [53], while two different studies reported that transcription of GAA repeats causes extensive hybrid RNA/DNA and RNA-loop formation, causing a premature RNA polymerase arrest [54,55]. Several studies demonstrated the existence of a very stable DNA triplex (also known as “sticky DNA”) formation in long GAA repeats that hampers proper *FXN* gene transcription [56,57,58,59]. As a result, FXN is expressed at much lower levels (5–30%) in FRDA patients as compared with healthy individuals [60].

Although a relatively low number of GAA repeats is present even in normal individuals (i.e., around 36 GAA repeats), due to the intrinsic instability of the repeats, 70–1700 repetitions have been found to be present in affected people [61,62]. It is worth highlighting that the length of these repeats correlates directly with disease progression and inversely with age of onset [63,64]. Indeed, patients in which the onset of the disease occurs at an older age with respect to what is considered the average pathologic occurrence (i.e., LOFA and vLOFA patients) have significantly shorter GAA expansions on both alleles when compared to individuals affected by typical FRDA [65]. In accordance with this, although the main cause of death in FRDA patients is cardiomyopathy, patients with delayed FRDA onset display only a mild ataxic phenotype associated with retained tendon reflexes and lower incidence of cerebellar atrophy, showing little or no extra-neurological signs such as cardiac complications, scoliosis, and foot deformities [10,66,67]. Given that triplet repeats could enhance heterochromatin formation and promote gene silencing, shorter GAA repeats could provide protection against more serious progression of the disease due to less-prominent epigenetic effects on *FXN* transcription [68].

Although FXN is ubiquitously expressed, its greatest expression in DRG, spinal cord, cerebellar dentate nuclei, cerebral cortex, pancreas, heart, liver, and skeletal muscle thereby reflects the typical pathological features that define FRDA disease [68,69,70,71]. The main transcript isoform, *FXN-1*, encodes a 210-amino-acid protein and is produced by the proteolytic processing of *FXN* immature transcript by the action of the mitochondrial peptidase (MPP) to include exon 1–5a. Additional transcripts have been reported, among which is *FXN-3* that differs from the main transcript for the presence of the alternative exon 5b [72] and encodes for a shorter protein of 171 amino acids due to the presence of an in-frame stop codon in its sequence [73]. The full-length 210-amino-acid isoform of the protein rapidly translocates from the cytosol to the mitochondria, where it is converted to the mature form by MPP activity [74]. Although many FXN functions are still poorly characterised, several studies demonstrated that this protein plays a pivotal role in the assembly of Fe-S clusters in the mitochondrial matrix [12,16], in heme synthesis [15], and in mitochondrial enzymes biogenesis [17]. In 1997, Babcock and colleagues discovered that deletion of the ortholog of *FXN* in yeast (Yfh1) causes iron accumulation in the mitochondria, demonstrating a role for FXN in iron homeostasis regulation [75]. Thereafter, a number of studies demonstrated that FXN is an iron-binding protein [76,77,78,79]. Moreover, FXN plays a role in DNA repair [80] and in cellular antioxidant defence signalling pathways [14]. In this regard, a recent study demonstrated that FXN is directly involved in the elimination of ROS species originated from the Cu^2+^- and Fe^3+^-catalysed degradation of ascorbic acid both in human and yeast cells [81]. Lack of FXN determines the reduction of total mitochondria number and also affects their morphology, leading to a consistent reduction of cristae and perturbing the overall mitochondrial organization [82]. The deficiency of FXN expression determines an abnormal functioning of mitochondrial enzymes and Fe-S cluster biosynthesis [83]. These defects result in reduced mitochondrial ATP production and in free iron accumulation [84], which induces a strong increase of Fenton-reactions-driven ROS production by interacting with oxygen moieties [29,85], thus increasing lipid peroxidation [86]. Iron accumulation, mitochondrial dysfunctions, lipid peroxidation, and reduction of glutathione peroxidase 4 (GPX4) activity or expression are distinctive hallmarks of ferroptosis, a recently discovered cell death mechanism [87]. Because all these impairments are also distinctive markers of FRDA, ferroptotic cell death has been proposed as one of the degenerative processes affecting cells deprived of FXN activity [88,89,90].

## 3. The NRF2 Signalling Pathway in FRDA

The cellular antioxidant response is directly regulated by NRF2 [91], a transcription factor belonging to the cap′n′collar (CNC) subfamily of basic-region leucine zipper (bZIP) family [92]. Following an oxidative insult and under metabolic imbalance, NRF2 translocates to the nucleus, where it binds to gene promoters containing specific enhancer sequences named antioxidant response elements (ARE) [93]. Structurally, NRF2 is composed of seven Nrf2-ECH homology domains (Neh1–7), each accomplishing distinct functions. The Neh1 domain is highly conserved across a wide range of species and is critical for DNA binding and association with its dimerization partners; it includes two highly conserved amino acid motifs, i.e., the DLG and ETGE motifs, which mediate the interaction with NRF2 negative regulator Kelch-like ECH-associated protein 1 (KEAP1), and seven lysine residues targeted for ubiquitination and subsequent proteasomal degradation of NRF2. The Neh3 displays transactivation activity and acts in concert with Neh4 and Neh5 to assure the expression of NRF2-target genes, while Neh6 and Neh7 are Keap1-independent negative regulators of NRF2 stability [94,95].

NRF2 has a rapid turnover, with a half-life of about 20–30 min [94]. Its expression is subjected to a tight regulation at various levels including transcriptional and post-transcriptional [94,96,97]. Upon binding of the Neh2 domain to its negative regulator KEAP1, NRF2 is sequestered into the cytoplasm and is tethered to actin bundles [98], which is a substrate adaptor protein for the Cullin 3-dependent E3 ubiquitin ligase complex (CUL3/RBX1). This facilitates the ubiquitination of seven lysine residues in the Neh2 domain of NRF2 and its proteasomal degradation [99]. Two models have been proposed to explain the KEAP1-mediated regulation of NRF2 stability. In the “hinge and latch” model, Keap1 interaction with the ETGE domain acts as a “hinge”, while a weaker interaction with the DLG motif acts as a “latch” [100]. Following an oxidative insult, a conformational change in KEAP1 causes its detachment from NRF2’s DLG motif, disturbing its ubiquitination and thus granting its stabilization and nuclear translocation [100]. In an alternative model, three critical KEAP1-reactive cysteine residues, namely Cys155, Cys273, and Cys288 [101], are modified after oxidative/electrophilic stresses, inhibiting ubiquitin conjugation to NRF2 by the KEAP1-Cul3 complex. This determines the disruption of KEAP1-Cul3 interaction, leading to the nuclear accumulation of de novo synthetised NRF2 [102]. While KEAP1 is the major cellular factor controlling NRF2 activation, the regulation of NRF2 protein stability also occurs via KEAP1-independent mechanisms. Similar to KEAP1, phosphorylation of NRF2 serine residues 335 and 338 by the action of the glycogen synthase kinase-3 beta (GSK3β) determines NRF2 polyubiquitylation and CUL3/RBX1-mediated degradation [103]. Lastly, the regulation of NRF2 protein expression is mediated by the action of the E3 ubiquitin ligase complexes βTrCP-S-phase kinase-associated protein-1 and HRD1, which target NRF2 for ubiquitination and proteasomal degradation through the interaction with its Neh6 and Neh7 domains [104]. The importance of these multiple and interconnected regulation mechanisms is outlined on the fact that NRF2 controls the expression of more than 1% of human genes that participate in the maintenance of basal cellular homeostasis and metabolic processes [103].

**Figure 2:**
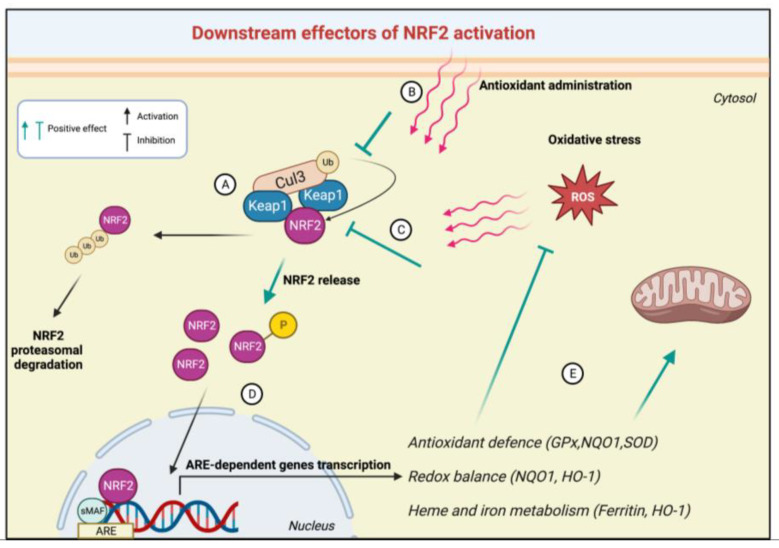
An overview of NRF2 pathway activation. (**A**) Under normal conditions, Cullin 3-dependent E3 ubiquitin ligase complex (Cul3) mediates the poly-ubiquitination of NRF2, leading to its proteasomal degradation. (**B**) Antioxidants or oxidative stress inhibit NRF2 ubiquitination and (**C**) disrupt KEAP1-NRF2 interaction, leading to NRF2 phosphorylation. (**D**) NRF2 migrates in the nuclear compartment, where it binds the sMAF proteins. (**E**) The NRF2–sMAF complex recognises ARE sequences promoting the transcription of NRF2-target genes.

Once in the nuclear compartment, leucine zipper domain-mediated heterodimerization with members of the small musculoaponeurotic fibrosarcoma (sMaf), including MafF, MafG, and MafK, makes NRF2 transcriptionally active. Indeed, the NRF2–sMaf complex recognises and binds the promoters of genes that contain specific cis-acting enhancer ARE sequences [105,106], promoting the transcription of a variety of antioxidant and phase II detoxification genes such as glutathione (*GSH*), NAD(P)H:quinone oxidoreductase (*NQO1*), heme oxygenase (*HO-1*), and superoxide dismutase (*SOD*) 1, 2, and 3 and many others [107,108,109]. In turn, NRF2-mediated expression of these genes ensures the maintenance of redox and metabolic homeostasis [110] and proper functioning of mitochondria [111] (Figure 2).

Upstream regulators of NRF2 expression are the transcription factors aryl hydrocarbon receptor (AhR) and NF-κB, which bind the *NRF2* promoter, leading to its transcription [112]. The presence of ARE-like sequences in the proximal region of *NRF2* promoter is involved in the positive regulation of its protein levels by NRF2’s own activity [113,114]. Conversely, NRF2 controls its own degradation by regulating the expression of the *KEAP1* gene by means of a feedback autoregulatory loop between KEAP1 and NRF2 [115].

Evidence indicates that NRF2 signalling is defective in FRDA cells and mouse models as well as in FRDA patients [20,21,22,23,24,25,26]. Several studies have demonstrated impaired NRF2 nuclear translocation, which is associated with decreased expression of its target genes, leading to increased sensitivity to oxidative stress and damage [20,116,117,118]. FRDA fibroblasts exhibit actin stress fibre glutathionylation (i.e., the covalent attachment of GSH moieties to cellular proteins) [119] and mislocalisation as a result of ROS accumulation generated by iron overload [20,119]. These alterations contribute to hamper NRF2 mobilization toward the nucleus [119,120]. FXN-silenced NSC34 neuroblastoma cells have been used to demonstrate reduced expression of Nrf2 as well as decreased expression of its target genes compared to control neurons [22]. Moreover, FXN deficiency caused decreased expression NRF2 and its target genes both in vitro in Schwann cells, fibroblasts, and HeLa cells and in vivo in the dorsal root ganglia and cerebellum of YG8R hemizygous mice, an FRDA mouse model that exhibits a mild, late-onset FRDA-like phenotype [21]. Of note, the same group demonstrated the presence of three highly conserved ARE sequences upstream of the transcription start site of the *FXN* gene [121]. After a screening of 1600 compounds, dyclonine, a topical anaesthetic also used to treat epilepsy [122], was found to rescue FXN deficiency in a dose-dependent manner in cells, animal models, and in FRDA patients [121]. The authors also demonstrated a dose-dependent stimulation of NRF2-target genes after treatment with dyclonine, which is associated with induced iron–sulphur cluster enzyme activity in animal and cell FRDA models [121].

## 4. NRF2 Activators: Antioxidant Therapeutic Approach to Mitigate Oxidative Stress in FRDA

Although a variety of antioxidants aimed at inducing NRF2 activity and/or expression have been tested since the 1990s, to date, there is conflicting evidence on the efficacy of different antioxidant treatments in FRDA. In the following section, we overview the beneficial effects of antioxidants as determined by preclinical research on FRDA and summarizse the main outcomes of different NRF2-inducing molecules’ administration. (Preclinical studies are summarised in Table 1 and clinical trials in Table 2.)

### 4.1. Natural Compounds

**Resveratrol** (RV) is a botanical phenol found in vegetables, tea, and wine, with anti-inflammatory [150], neuroprotective [151], anti-cancer [152], and cardioprotective proprieties [153]. Its beneficial effects rely on the ability of RV to neutralise ROS either directly, as a scavenger, or indirectly through the upregulation of the expression of cellular defence-related genes [154]. Its antioxidant scavenging activity is associated with the presence of phenolic rings with three hydroxyl groups, which allows the transformation of free radicals [155]. RV has been shown to stimulate NRF2 phosphorylation through a phosphatidylinositol 3-kinase/protein kinase B (PI3K/Akt)-dependent mechanism [156]. Furthermore, RV exerts its antioxidant activity through the direct involvement of sirtuin 1 (SIRT1) [157], an enzyme belonging to class III histone deacetylase family that regulates various cellular processes including gene expression, DNA repair, metabolism, oxidative stress response, and mitochondrial function and biogenesis [158]. SIRT1 appears to interact with the NRF2/KEAP1/ARE pathway in a bidirectional manner. On the one hand, it has been shown that SIRT1 significantly enhances the activity of ARE-dependent transcription by decreasing KEAP1 expression, promoting NRF2 nuclear translocation and ARE-binding ability/transcriptional activity, and augmenting the protein levels of HO-1. Meanwhile, NRF2 positively regulates SIRT1 protein expression via direct binding [159].

A variety of studies have demonstrated both in vitro and in vivo that RV intake ameliorates oxidative stress through NRF2 induction, diminishes lipid peroxidation, and increases the activity of Mn-SOD via NRF2/HO-1 signalling pathway [160,161,162]. A recent study evaluated the ability of eight individual drugs administration to induce *FXN* transcription and mitochondrial biogenesis both in vitro using FRDA patient-derived fibroblasts and in vivo exploiting YG8LR mice [123]. RV and dimethyl fumarate (DMF) were found to increase both *FXN* transcription and mitochondrial biogenesis in these cells. The authors also tested the simultaneous administration of both compounds in cerebellar granule neurons (CGNs) and cardiomyocytes from FRDA mouse models, demonstrating the synergic effect of RV and DMF in terms of *FXN* mRNA expression, mitobiogenesis increase, and ROS reduction. Further, to corroborate their findings in vivo, YG8LR FRDA mice were subjected to the two following treatments. The short-term treatment was based on intraperitoneal injection for 5 days of either vehicle (PBS/5% Tween 20/5% PEG 400/2% DMSO) or drug as follows: (i) 10 mg/kg/d DMF, (ii) 10 mg/kg/d RV, or (iii) 10 mg/kg/d DMF and 10 mg/kg/d RV combined. A second long-term treatment consisted of oral gavage administration three times per week for 5 months of either vehicle or 200 mg/kg/d DMF and 200 mg/kg/d RV combined, a concentration equivalent to that given to humans but adjusted to mouse weight. While the first treatment modality determined a slight increase of *FXN* mRNA and mitobiogenesis (as measured by mitochondrial DNA/nuclear DNA ratio), which did not reach statistical significance and yielded no improvement in behavioural tests, the second determined an improvement of motor function as assessed by rotarod performance test [123].

With regard to clinical trials, in an open-label study on 24 FRDA patients, the effect of oral administration of mega resveratrol, a pharmaceutical-grade, 99% pure trans-resveratrol made of high absorption micronised resveratrol (Danbury, CT), was evaluated [131]. Both high-dose (5 g/d) and low-dose (1 g/d) RV were assessed over a 12-week period, and participants under other antioxidants therapies underwent a 30-day washout prior to enrolment to ensure a specific effect of RV. Despite the significant neurologic, audiologic, and speech improvements found in the high-dose group, no effects on FXN levels or in the cardiac outcomes were detected in patients independently from the dose administered [131].

**Coenzyme Q_10_** (CoQ_10_) is an essential constituent of the mitochondrial respiratory chain that carries electron from complexes I and II to complex III [163]. It is the only endogenously synthesised lipid-soluble antioxidant that is also involved in antioxidant cell defences preventing oxidation of proteins, lipids, lipoproteins, and DNA [9]. CoQ_10_ has been reported to decrease oxidative stress by acting as an antioxidant and free-radical scavenger [164].

In vitro, CoQ_10_ was found to inhibit DMN-induced liver fibrosis in H4IIE hepatoma cells through NRF2/ARE activation, whereas its protective effects were abolished in Nrf2-null MEF cells [165]. The protective effects of CoQ_10_ were also reported in the rat PC12 pheochromocytoma cell line, with particular reference to protection from neurotoxicity caused by H_2_O_2_ via transcriptional activation of Nrf2 and upregulation of antioxidant enzyme activity [166]. Consistently, CoQ_10_ supplementation has been shown to increase Nrf2 and HO-1 protein expression levels in rats subjected to chronic exercise training [167].

Evidence regarding FRDA in vitro studies showed that MitoQ, a derivative of CoQ_10_, was able to reduce cell death originating from endogenous GSH depletion in human fibroblasts obtained from FRDA patients [124]. Other studies also demonstrated defective CoQ_10_ synthesis [168] and lower serum levels in FRDA patients compared to healthy individuals [132].

CoQ_10_ treatment has also been evaluated in various clinical trials in synergic administration with vitamin E, yielding an improvement of cellular bioenergetics as measured in vivo in the cardiac and skeletal muscle of 10 FRDA patients after a six-month treatment [133]. These results were strengthened by a study in which 10 FRDA patients were enrolled over a longer period (47 months) [169]. A CoQ_10_/vitamin E trial was started in 2008, involving 50 FRDA patients randomly assigned to high- or low-dose group, and was carried over a two-year period [132]. It should be noted that the low-dose administration group was handled as the control group due to the difficulty in recruiting healthy control groups. However, no changes in ICARS scores were observed, thus indicating no benefits or differences between high- and low-dose therapy. Conversely, a post hoc analysis revealed a robust improvement in ICARS scores in 16 patients, and cross-sectional natural-history data analysis suggested that half of the patients benefited from CoQ_10_/vitamin E treatment, presenting a modest improvement and a slower progression of symptoms with respect to the predicted worsening that is typical of FRDA patients [132].

**Acetyl-l-carnitine** (ALCAR) is the principal acetyl ester of L-carnitine, an amino acid that transports activated long-chain fatty acids into the mitochondria, where they are degraded by β-oxidation, thus boosting energy metabolism [170]. Moreover, its neuroprotective, anti-inflammatory, and antioxidant effects have been established in several models of neurodegenerative disorders, including degenerative cerebellar ataxia [134], Parkinson’s disease (PD) [171], and Huntington’s disease [172]. Of note, it has been demonstrated that ALCAR activates the NRF2 pathway by inducing Keap1 acetylation [173,174]. Consequently, treatment with ALCAR has been found to induce HO-1 expression in a time- and dose dependent-manner in rat type I astrocytes [173] and an increase of antioxidant protein and transcript levels of CAT, SOD, GPX, NRF2, KEAP1, and GSH in human lens epithelial cells (HLECs) [174].

Conflicting results are present in the literature regarding the treatment of FRDA patients with ALCAR. In order to rescue mitochondrial dysfunction, a double-blind, placebo, crossover trial tested the efficacy of L-carnitine and creatine in 16 FRDA patients by evaluating changes in ICARS scores and in echocardiographic profile. Unfortunately, neither significant improvements in ICARS scores nor changes in echocardiographic parameters were found [175]. Conversely, a double-blind, crossover, placebo-controlled clinical trial with ALCAR in patients with degenerative cerebellar ataxias, including 11 FRDA-affected patients, showed improvements in coordination after 3 and 6 months and a significant beneficial effect on muscle tone after 6 months [134].

**Sulforaphane** (SFN) is a phytocompound abundant in cruciferous vegetables (e.g., broccoli, Brussels sprouts) that exhibits neuroprotective, cardioprotective antioxidant, anti-inflammatory, and antiapoptotic effects [176]. Furthermore, SFN stimulates neurogenesis, increasing brain-derived neurotrophic factor (BDNF) and wingless-type (WNT) protein levels [177]. Its antioxidant activity due to the iso-thiocyanate (ITC) group along with the proprieties mentioned above have granted SFN wide use in preclinical studies for a broad range of human neurological diseases such as Alzheimer’s disease (AD), PD, and multiple sclerosis (MS) [177,178,179,180]. Of note, SFN exerts its therapeutic effect by activating multiple cellular mechanisms including NRF2-mediated induction of phase 2 detoxification enzymes, namely NQO1, HO-1, and GCL [126]. In addition, it was shown that the combined treatment of a Nrf2-knockout mouse model with SFN and inhibitors of gamma-glutamylcysteine synthetase (GCS), i.e., one of the enzymes involved in GSH synthesis [181], abolished the neuroprotective effects of SFN, thus confirming that SFN targets NRF2. Indeed, SFN administration was found to reinforce NRF2 binding to ARE-provided gene sequences in a dose-dependent manner [182]. As a consequence of its electrophilic nature, SFN can also interact with thiol groups of many different proteins including KEAP1, thereby preventing NRF2 ubiquitination by the modification of several cysteines and suppressing of GSK3β activity, ultimately leading to increased NRF2 stabilization and nuclear translocation [183,184]. In line with the aforementioned proprieties, several in vitro studies on FRDA demonstrated a promising beneficial effect of SFN [25,125,126]. To analyse the effect of SFN and deuterised 4 poly-unsaturated fatty acid (d4-PUFA) on lipid peroxidation and mitochondrial dysfunction, fibroblasts derived from two FRDA mouse models, namely YG8R and KIKO, were used [125]. SFN administration was found to protect from oxidative stress, preventing lipid peroxidation and cell death [125]. One study exploited neural stem cells (NSCs) isolated from FXN KIKO mice, which display FRDA-phenotypic defects at very early stages of neurogenesis, as indicated by abnormal NSC proliferation and differentiation. This defect is associated with impaired NRF2 mRNA and protein expression that, in turn, causes a reduction of NQO1 and HO-1 expression, which are two master NRF2-target genes. Notably, treatment with SFN has been found to restore a proper differentiation program in NSCs and to rescue NRF2, NQO1, and HO-1 mRNA and protein levels [25].

Moreover, SFN treatment of FXN-silenced NSC34 motor neurons has proven to restore FXN protein expression along with NRF2 protein and mRNA expression and the subsequent activation of its target genes [24]. Accordingly, axonal re-growth and increased neurite numbers strengthened the promising effects of this molecule [24], which were fully confirmed in FRDA patient-derived fibroblasts demonstrating that SFN-induced NRF2 activation was able to promote *FXN* mRNA increase [126].

Despite these promising results, data of SFN administration, if any, in clinical trials of FRDA are lacking, although beneficial effects of SFN in other neurologic disorders such as schizophrenia [185] and autism spectrum disorder (ASD) have been described [186,187].

**Dimethyl fumarate** (DMF) is the methyl ester of fumaric acid and, like SFN, is an established NRF2 activator, although precise mechanisms underlying this action 1remain elusive [188,189]. One study suggested that DMF directly interacts with NRF2 by promoting a covalent modification of its DNA binding domain [190], but how DMF induces this conformational change in NRF2 is not yet fully understood. Other studies reported that under oxidative stress, DMF significantly increases the expression of Nrf2-target genes, including *SOD2* and *NQO1*, among others, in rat neural stem cells [191]. The effect of DMF in FRDA has been evaluated in lymphoblasts isolated from the YG8R mouse model and in DMF-treated lymphocytes derived from FRDA patients, showing that DMF increases *FXN* mRNA levels by 93% in FRDA lymphoblasts and by 52% in mice in vivo. Furthermore, DMF has been found to increase mitochondrial biogenesis and to reduce the R-loop found at GAA expanded sites, as evaluated in blood lymphocytes from patients in a dose-dependent manner [127]. Because of its electrophilic activity, DMF can non-specifically covalently modify cysteine thiols, producing severe systemic side effects [191,192]. For this reason, a study undertaken to assess DMF safety established the maximum effective (110 mg/d) and tolerated (160 mg/d) doses in the FXNKD mouse model, a doxycycline-inducible model of FRDA [192]. These doses were found to overlap the currently approved human-equivalent doses of DMF for the treatment of MS (480 mg/day) and psoriasis (720 mg/day) [128]. Moreover, the authors of this study demonstrated that DMF rescues the activity of brain mitochondria-related enzymes (e.g., Complex II and Complex IV of the respiratory chain and aconitase) [128].

Similar to SFN, DMF has not yet been tested in clinical trials for the treatment of FRDA patients. However, DMF is currently under clinical investigation to treat relapsing forms of MS [193].

**Curcumin** is a natural polyphenol found in the rhizome of *Curcuma longa* (turmeric), which gained attention in the contest of neurodegenerative diseases due to its antioxidant, anti-inflammatory, iron chelation, and anti-tumour activities [194]. It has been found to confer neuroprotection in AD, PD, and other related neurodegenerative and neuropsychiatric disorders [195,196]. Curcumin has been shown to activate the NRF2/Keap1 pathway both in vitro and in vivo [197]. Recent evidence demonstrated that curcumin is able to stimulate the activation of NRF2 pathway in vitro by inhibiting KEAP1, positively influencing the NRF2 expression, and by improving its nuclear translocation [197,198]. In the same vein, in vivo curcumin treatment has been found to enhance *Nrf2* mRNA expression (1.5 fold) and HO-1 mRNA expression (9.5 fold) along with phase-II enzymes’ [199,200]. Consistently, it has been also demonstrated that *Nrf2* knockdown abolished curcumin’s positive effect both in vitro and in vivo [201]. Despite these positive effects, curcumin bioavailability is very limited due to poor water solubility and absorption through the gastrointestinal tracts [202]. In order to enhance its bioavailability, modified curcumin forms such as curcumin hydrogel [203] and curcumin graphene [204] have been synthesised. Of particular importance, a recent study demonstrated that loading curcumin in silk fibroin (SF) to form nanoparticles (NPs) (Cur@SF NPs) not only eliminates iron from the heart and ameliorates oxidative stress in general in fibroblasts derived from FRDA patients but also potentiates Fe-S cluster biogenesis in tissue from YG8R FRDA mice. Moreover, Cur@SF NPs showed a significant advantage in neuron and myocardial function, thereby improving FRDA mouse behaviour scores [129]. These data suggest that Cur@SF NPs has promising therapeutic potential for the treatment of FRDA.

### 4.2. Synthetic Compounds

**Idebenone** (IDE) is a synthetic compound that is a structural analogue of ubiquinone (the oxidised form of CoQ_10_), with enhanced bioavailability because of lower molecular weight and improved water solubility [26]. In vitro, IDE, displays vigorous antioxidant activity by facilitating electrons flux through the mitochondrial electron transport chain, thus leading to increased production of ATP [205,206]. Therefore, IDE has been used in mitochondria-related diseases such as Leber hereditary optic neuropathy (LHON) [207,208] and for the treatment of hereditary myopathies [209]. IDE was shown to activate the NRF2 signalling pathway by promoting its nuclear translocation after H_2_O_2_ treatment by using an in vitro model of a human retinal pigment epithelium (RPE) cell line [210]. Notably, it has been demonstrated that IDE can also activate NRF2 and the transcription of its target genes in vitro in fibroblasts obtained from skin biopsies of FRDA patients [126]. For these reasons, in the past decades, IDE has gained attention in the context of clinical trials involving FRDA patients, although with some degree of variability observed among treatment duration, number of patients, and neurological and cardiac outcomes. In fact, improvement in cardiac function and reduction of cardiac hypertrophy were found in the majority of clinical trials [135,136,137], although some exceptions are present [138,139]. However, these effects were not accompanied by any improvement in neurological parameters except for one study, where improvements in fine movements were demonstrated [135], and for three trials reporting a reduction of the progression of cerebellar manifestations by ICARS and FARS scores but no improvement of heart hypertrophy [138,139,140,211]. Two recent trials corroborated the discordant results obtained from previous investigations [141,142]. In fact, one reported no significant differences on assessment of treatment between groups [141], although this study was performed during a very short period (2 months). The second trial was aimed at evaluating different dosages of IDE during a long-term follow up that ranged from 4 to 11 years in 27 FRDA patients [142]. Sadly though, large interindividual and intraindividual variability was observed in plasma IDE concentrations, associated with no positive consistent effect [142].

Two studies evaluated the use of a combination of drugs to simultaneously target different aspects of this pathology. The first reported that the synergic treatment with both IDE and erythropoietin (EPO), a glycoprotein hormone able to increase *FXN* mRNA levels [211], was well tolerated and safe, although it resulted in no significant hematologic, clinical, or biochemical beneficial effects [143]. The second analysed the effect of 5 mg/kg/d tocotrienol supplementation for one year in FRDA patients who were already under IDE treatment [144]. This idea was fuelled by the observation that this antioxidant compound belonging to the vitamin E family specifically enhances *FXN-3* mRNA expression (3.49-fold) in mononuclear blood cells derived from FRDA patients, while no effects were seen in *FXN*-*1* transcription [73]. However, the effect on patients was not enthusiastic, although low-dose tocotrienol supplementation decreased oxidative stress indexes after two months’ supplementation. Unfortunately, the effect of one-year supplementation results was not acquired due to technical problems [144].

**EPI-743**, also known as vatiquinone, is an orally bioavailable, synthetic vitamin E analogue designed for inherited mitochondrial diseases [212]. This drug can be administered orally, is safe and well tolerated, and importantly, it crosses the blood–brain barrier (BBB) [145]. By using fibroblasts isolated from DNA-polymerase-γ-deficiency patients, characterised by the lack of the key enzyme responsible for mitochondrial DNA replication and repair [213], it was shown that EPI-743 treatment positively regulated the expression of *HO-1*, *NQO1*, and genes related to GSH synthesis [145], which are renowned targets of NRF2 transcriptional activity [107].

In fibroblasts obtained from skin biopsies of FRDA patients, EPI-743 treatment was found to significantly increase *FXN* gene expression [126]. Furthermore, the authors demonstrated strong NRF2 activation at both the transcript and protein levels, coupled to a significant induction of its target genes, NQO1 and γ-glutamylcysteine ligase (GCL) [126]. Accordingly, EPI-743 treatment of FRDA-derived fibroblasts and FXN-silenced mouse C_2_C_12_ myoblasts confirmed Nrf2 nuclear translocation and activation of its downstream targets, as indicated by the rescue of mitochondrial tubular network and the potentiation of cellular antioxidant defence with, among others, GPX4 enzyme expression increase. This effect, along with the parallel reduction of lipid peroxidation, pointed at the NRF2 signalling axis as a key pathway in regulating cellular processes that oppose ferroptosis, leading to two clinical trials involving EPI-743 treatment. In 2016, the effect of EPI-743 in FRDA patients carrying an expanded GAA in one allele and a point mutation on the other one was assessed, yielding significant neurological improvements with no side effects over a period of 18 months. Of note, improvements in all FARS subscales were found, with particular reference to bulbar and upper-limb coordination upon EPI-743 administration [146]. On the other hand, in a double-blind, randomised, and controlled trial, 6-month EPI-743 treatment showed no significant improvements in the primary or secondary FARS score outcomes. However, by using a longitudinal modelling, at 24 months, EPI-743 treatment was associated with significant improvement of neurological function, as indicated by FARS-neuro scores (1.8 points) and disease progression, with no drug-related serious adverse events or dose-limiting toxicities [147].

Currently, recruitment for an open-label study to evaluate the efficacy of EPI-743 on FRDA patients younger than 7 years is ongoing (https://clinicaltrials.gov/ct2/show/NCT05485987, accessed on 1 March 2023).

**Figure 3:**
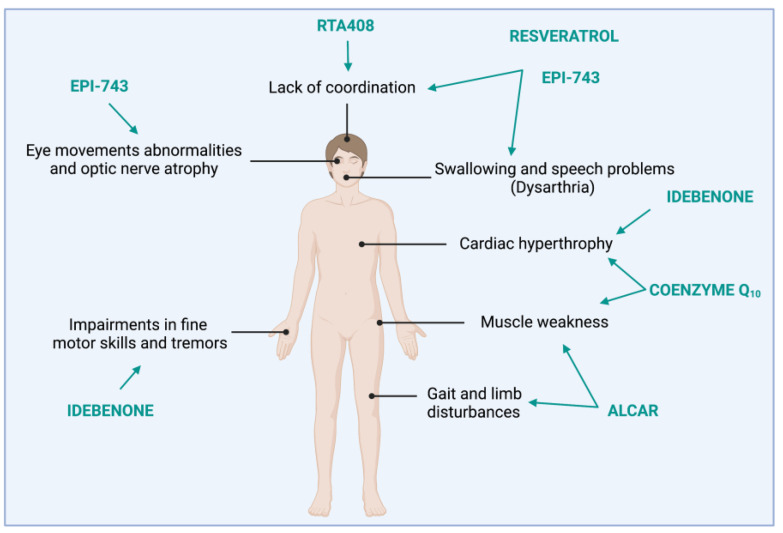
A summary of clinical defects of FRDA patients and the effect of antioxidant administration. Created with @BioRender.

**Omaveloxolone** (RTA408) is a synthetic triterpenoid compound with anticancer, anti-inflammatory, and antioxidant activity [214]. RTA408 has been developed to activate NRF2 signalling, and it is being investigated in clinical trials for the treatment of patients with FRDA as a means to counteract mitochondrial dysfunction, sensitivity to oxidative stress, and impaired mitochondrial ATP production. In particular, it has been demonstrated that RTA408 inhibits KEAP1 by directly binding to its Cys151, thus leading to NRF2 stabilization and preventing its ubiquitination. This thereby improves mitochondrial function and reduces oxidative stress [214]. Preclinical studies using FRDA human fibroblasts, KIKO, and YG8R mouse FRDA models demonstrated that RTA408 is able to prevent lipid peroxidation and fibroblast cell death after induction of oxidative stress and to promote mitochondrial respiration [130]. One of the effects of FXN decrease is the disruption in the maintenance of mitochondrial membrane potential (ΔΨm), which is considered a marker of mitochondrial health [215]. RTA408 administration prior to exposure to an oxidative insult maintains ΔΨm within physiological values, indicating a positive effect of RTA408 on mitochondrial function [130]. In fibroblasts derived from FRDA patients, RTA408 exerts this effect by increasing NRF2 mRNA and protein expression and the subsequent induction of NRF2-target genes (i.e., NQO1, HO-1, and GCL), with no significant changes in *FXN* mRNA expression [126]. In 2018, safety, pharmacodynamics, and the potential beneficial effect of various doses of RTA408 in FRDA patients were evaluated, demonstrating that, in addition to being safe and well tolerated, a 160 mg/day administration resulted in significant improvements in neurological function as measured by modified (m)FARS score (a simplified version of the FARS score) (Figure 3). Moreover, this treatment determined an increase in indirect NRF2-target genes expression, such as ferritin and aspartate amino transferase (AST) [148]. These results were confirmed in the second part of this randomised, placebo-controlled, double-blind study, where RTA408 was found to be effective in improving bulbar, upper- and lower-limb coordination, and upright stability as estimated by the mFARS scores, even at a lower dose (150 mg/day) [149]. The third part of the trial, which is the extension phase, is estimated to be completed in December 2024 and will provide useful hints on the long-term safety and tolerability of RTA 408 in qualified patients who completed part 1 or part 2. (https://clinicaltrials.gov/ct2/show/NCT02255435, accessed on 1 March 2023).

## 5. Constraints of Current Therapeutic Approaches and Future Prospects

The precise sequence of pathogenic events in FRDA remains uncertain, and to date, no cure for FRDA is approved by the Food and Drug Administration (FDA) [216]. Current therapeutic approaches pursue two main objectives: (i) to augment or restore FXN expression both by pharmacological interventions [217] and by gene therapy [218] or (ii) to modulate the downstream processes responsible for altered mitochondrial metabolism by reducing ROS production or by increasing NRF2 activation and/or expression. The latter approach is particularly significant given that the lack of a proper NRF2-regulated antioxidant response contributes to the progression of this fatal disease [20,21,22,23,24,25,26]. Most of the promising preclinical data on antioxidants-based therapy, aimed at restoring or inducing the activation of NRF2 pathway, failed to show significant improvements when translated to clinical evaluation [131,181,194]. The debate aimed at clarifying these conflicting and likely not conclusive results is still open. Nevertheless, we may attempt to identify reasons that underlie the reduced clinical efficacy of these strategies: (i) The NRF2 activator family includes an extremely variegated group of molecules [219], most of which are electrophilic compounds with peculiar chemical structures that can influence their bioavailability. In fact, while this parameter can be easily controlled through in vitro systems, it thereby hampers in vivo treatments. As an example, trials with compounds such as L-carnitine provided no significant outcomes due to its poor bioavailability [175]. Conversely, studies performed with a more bioavailable derivative compound, ALCAR, the principal acetyl ester of L-carnitine, significantly improved motor coordination and patients’ muscle tone [134]. In the same way, other molecules such as curcumin are poorly absorbed by the intestinal epithelium [202]. In this regard, nanoparticle-based delivery methods seem to be a promising tool to hijack this issue [203]. (ii) A fundamental matter is that the response to treatments varies between animal models, typically rodents, and the human model. In this regard, a systematic review that analysed the concordance between animal experiments and clinical trials [220] led authors to conclude that promising beneficial effects of a given treatment in animal models very rarely translate to human trials. A particular emphasis was given to the relevance of selecting animal models that recapitulate the human disease in order to avoid misleading results that could be linked to differences between human and mice [220]. This is particularly tricky in most of the studies using FRDA animal models, which did not fully reflect the human disease [221]. For instance, the available FRDA mouse models mainly manifest neurologic or cardiac symptoms in a mutually exclusive manner [222]. Animals providing both, such as the neuron-specific enolase (NSE) mutants, display a severe phenotype that results in a short lifespan [222]. The KIKO mouse model was generated [223], introducing a (GAA)_230_ repeat expansion into the first intron of the mouse *FXN* locus, mimicking the genetic defect that occurs in 96% of cases of FRDA disease [11]. Although KIKO mice only express a 25–36% residual FXN protein expression, they display no iron deposits and only mild signs of fibrosis in the heart, along with no coordination defects [221]. These phenotypic differences certainly contribute to provide incomplete information about the potential efficacy of a given administered antioxidant drug. Recently, a mouse model named YG8–800, developed by Jackson Laboratories Inc. (Farmington, CT, USA), was described by Gérard and colleagues to accurately reflect the human disease with low expression of FXN (20% compared to control), a progressive neuromuscular degeneration, and the beginning of heart hypertrophy at 26 weeks [224]. Thus, this model could represent a promising resource for further preclinical studies involving FRDA. (iii) The number of participants in FRDA clinical trials is another issue, as it has been quite small in the majority of the clinical trials undertaken up to now [134,135,140,145] or was limited to rare variant group analysis [146]. As FRDA is a rare disease characterised by some degree of variability between patients’ groups [86], it can be expected that the number of patients available to participate in clinical trials is reduced. This is a factor of primary importance considering that small patient group samples limit the power of the study, not reaching the significant statistical threshold, as we have seen for several of the trials examined [141,175,187]. In the same way, treatment duration should be also taken into account. Indeed, clinical studies have often shown to be brief and leading to uncertain conclusions regarding significant and lasting benefits [133,134,135,138,139]. This aspect is of the utmost importance in FRDA treatment considering that the major endpoints used to analyse FRDA progression, such as FARS and SARA scores, show greater sensitivity to change over 2 years than over 1 year [225], while treatment duration of antioxidants-based trials did not span the timing of the overall set of tests [133,134,135,138,139]. (iv) The age of the enrolled patients and the pathologic onset also could represent an issue. In fact, although FRDA symptoms usually manifest at around 10 ten years of age in patients, lack of FXN can be present since development, and the literature evidence has demonstrated pre-symptomatic neurodevelopmental defects, along with Nrf2 impairments [25,226,227], suggesting that managing FRDA impairments as soon as possible could lead to more favourable outcomes. In line with this, one clinical trial with IDE treatment for both paediatric and adult FRDA patients revealed the most significant neurological benefits within the paediatric group [137], underlying that the age at which treatment is initiated may be an important factor in the efficacy of a given therapy against FRDA progression.

## 6. Conclusions

The issues discussed here indicate that there are still many dark spots that require elucidation in order for FRDA patients to benefit from effective antioxidant therapy. However, although the relationship between the lack of FXN in FRDA and the benefits of NRF2–ARE axis activation have not yet been fully elucidated, it is important to highlight the significant correlation found between FXN expression and NRF2 activity [21], which is associated with the presence of three highly conserved ARE sequences on the *FXN* gene promoter, which are crucial for the binding of the NRF2–sMaf complex to promote the transcription of detoxification and antioxidant genes [105,106]. In light of this, by addressing the limitations that currently separate pre-clinical models from patient trials and improving early diagnostic systems, it is auspicial that new treatments or molecules, especially in conjunction with other therapeutic approaches (i.e., lentivirus-mediated *FXN* gene delivery [228] or human embryonic stem cell (hESC) therapy [229]), may pave the way for effective treatments of this pathology.

## Figures and Tables

**Table 1:** Therapeutic targeting of NRF2 activators in preclinical trials.

Compound	Mode of Action	Model	Dosage	Effect	Ref.
**RESVERATROL**	Induction of NRF2 phosphorylation (PI3K/Akt-dependent mechanism)	In vitro Fibroblasts derived from FRDA patients; Cardiomyocytes and cerebellar granule neurons	100 μM RV/30 μM DMF	Increase in *FXN* mRNA transcription and mitochondrial biogenesis	[123]
	Promotion of SIRT1 activation, which leads to reduced KEAP1 expression and increased NRF2 nuclear translocation	In vivo YG8LR mice	200 mg/kg/d RV + DMF	Decrease in ROS levels; Improvement in rotarod performance	
**COENZYME Q_10_**	Inhibition of NRF2 proteasomal degradation by promoting its stabilization and nuclear translocation	In vitro Fibroblasts derived from FRDA patients	0.1 pM–50 μM	Prevention of cell death in GSH-depleted cells	[124]
**SULFORAPHANE**	Prevention of NRF2 proteasomal degradation by interaction with KEAP1, thereby promoting NRF2; Nuclear translocation and reinforcing NRF2 binding to ARE sequences	In vitro KIKO and YG8R mice fibroblasts	50 nM	Prevention of lipid peroxidation and cell death	[125]
		Frataxin-silenced NSC34 motor neurons	5 μM	Increase in FXN protein; Increase in Nrf2 transcript and protein expressions; Increase in NQO1, SOD, and GSH content; Axonal re-growth and increased neurites’ numbers	[24]
		Fibroblasts derived from FRDA patients	10 μM	Increase in NRF2 transcript and protein expressions; Increase in NRF2-target genes (*NQO1* and *HO-1*) expressions	[126]
		Neural Stem Cells from KIKO mice	5 μM	Reduction of ROS levels; Re-establishment of a proper differentiation program	[25]
**DMF**	Promotion of covalent modification of NRF2 DNA binding domain, leading to NRF2 activation	In vitro Lymphocytes derived from FRDA patients	10–30 μM	Increase in *FXN* mRNA and protein expression and mitochondrial biogenesis; Reduce of R-loop at GAA sites in FRDA patients	[127]
		In vivo YG8R mouse model	5 and 10 mg/kg	Increase in FXN mRNA and protein expression	
		FXNKD mouse model of FRDA	110–160 mg/day	Rescue of brain mitochondria-related enzymes (Complex II, Complex IV, and aconitase)	[128]
**CURCUMIN**	Inhibition of NRF2 proteasomal degradation by promoting its stabilization and nuclear translocation	In vivo YG8R FRDA mice	150 mg/kg 5 days	Increase in Fe-S biogenesis; Elimination of iron deposits from heart	[129]
**IDEBENONE**	Inhibition of NRF2 proteasomal degradation by promoting its stabilization and nuclear translocation	In vitro Fibroblasts derived from FRDA patients	1 μM	Increase in Nrf2 transcript and protein expression; Increase in NQO1 (Nrf2-target gene) transcript expression	[126]
**EPI-743**	Inhibition of NRF2 proteasomal degradation by promoting its stabilization and nuclear translocation	In vitro Fibroblasts derived from FRDA patients	1 μM	Increase in *FXN* mRNA andNrf2 transcript and protein expression;	[126]
			1 μM	Increase in Nrf2 nuclear translocation;Rescue of mitochondrial tubular network	[118]
**RTA408**	Inhibition of KEAP1 by direct binding to its Cys151	In vitro KIKO and YG8R mice cerebellar granule neurons (CGNs)	50 nM	Restoration of OXPHOS complex; Prevention of lipid peroxidation; Reduction of mROS and increase in GSH content	[130]
		Fibroblasts derived from FRDA patients	50 nM		
		Fibroblasts derived from FRDA patients	100 nM	Increase in Nrf2 transcript and protein expression; Increase in NQO1, GCL, and HO-1 (Nrf2-target genes) transcript expressions and in GSH content	[126]

**Table 2:** Therapeutic targeting of NRF2 activators in clinical trials.

**Compound**	**Mode of Action**	**Patients**	**Dosage and Time**	**Effect**	**Ref.**
**RESVERATROL**	Induction of NRF2 phosphorylation (PI3K/Akt-dependent mechanism); Promotion of SIRT1 activation, which leads to reduced KEAP1 expression and increased NRF2 nuclear translocation	24	1 or 5 g/daily	Significant neurologic, audiologic, and speech improvements in the high-dose group	[131]
			12 months	No improvement in cardiac outcomes or *FXN* expression	
**COENZYME Q_10_**	Inhibition of NRF2 proteasomal degradation by promoting its stabilization and nuclear translocation	43	CoQ_10:_ 600 mg/d (2× 100 mg for three times/day) + Vitamin E 2100 IU/day supplementation	Restoration of CoQ_10_ serum levels	[132]
		10	2 years CoQ_10:_ 400 mg/d + Vitamin E 2100 IU/day supplementation 6 months	Improvement in cardiac and skeletal muscle bioenergetics; Improvements in ICARS score (post hoc analysis) in 49% of patients	[133]
**ALCAR**	Prevention of NRF2 proteasomal degradation by interaction with KEAP1, NRF2 nuclear translocation, and reinforcement of NRF2 binding to ARE sequences	11	1000 mg/d twice a day 6 months	Improvements in coordination after 3 and 6 months and significant effect on muscle tone after 6 months	[134]
**IDEBENONE**	Promotion of covalent modification of NRF2 DNA binding domain, leading to NRF2 activation	3	5 mg/kg/d 4–9 months	Decrease in myocardial hypertrophy; Improvements in fine movements	[135]
		8	5–20 mg/kg/d 3–5 years	Significant reduction of cardiac hypertrophy in six of eight patients.	[136]
		24 (10 paediatric, 14 adults)	5–20 mg/kg/d 3–5 years	Prevention of progression of cardiomyopathy in both paediatric and adult patients; Stabilizing effect on neurological dysfunction only in paediatric patients	[137]
		70	10–54 mg/kg/d 6 months	No improvements in neurological outcomes and no assessment of cardiac outcomes	[138]
		70	450/900 mg/d or 1350/2250 mg/d 6 months	No decrease in hypertrophy or improved cardiac function	[139]
		9	5 mg/kg/d 1 year	Cerebellar improvement (after 3 months); Significant reduction of ICARS scores	[140]
		29	1350–2250 mg/d 2 months	No improvements in ICARS score or in cardiac outcomes	[141]
		27	5–20 mg/kg/d 4–11 years	No improvements in neurologic or cardiac outcomes	[142]
**IDEBENONE +Erythropoietin**	Erythropoietin: increases *FXN* mRNA levels	16	IDE 5 mg/kg/d EPO: 20,000–40,000 IU	No significant hematologic, clinical, or biochemical impact	[143]
**IDEBENONE** **+Tocotrienol**	Tocotrienol: enhances *FXN-3* mRNA expression	14	IDE + Tocotrienol mixture 5 mg/kg/d 2 months (expected 1 year)	Decrease in oxidative stress indexes (GSH/GSSG ratio; carbonyl group)	[144]
**EPI-743**	Inhibition of NRF2 proteasomal degradation by promoting its stabilization and nuclear translocation	14 (2 FRDA patients)	100 mg, two times per day, increased to three times per day on day 29, 12 weeks	Improved strength, exercise tolerance, speech fluency, sleep, increased social interaction; Partial rescue of complete cortical blindness	[145]
		3 FRDA patients (rare variant)	400 mg 18 months	Significant improvement in neurological functions (FARS score) already at 6 months	[146]
		63	200–400 mg 2 years	No improvements in visual acuity, 25-foot walk test, peg-hole test, or echocardiography; Post hoc analysis showed significant improvement in FARS score	[147]
**RTA408**	Inhibition of KEAP1 by direct binding to its Cys151	69	160 mg/d/12 weeks	Significant improvements in FARS score; Increase in Nrf2-target gene expression.	[148]
		155	150 mg/d/48 weeks	Second part of the trial: results confirmed even at a lower dosage	[149]
		Extension phase		Estimated to be completed in December 2024	NCT02255435

## Data Availability

Not applicable.
